# Bioprosthetic Aortic Valve Degeneration: a Review from a Basic Science Perspective

**DOI:** 10.21470/1678-9741-2020-0635

**Published:** 2022

**Authors:** Tiago R Velho, Rafael Maniés Pereira, Frederico Fernandes, Nuno Carvalho Guerra, Ricardo Ferreira, Ângelo Nobre

**Affiliations:** 1 Cardiothoracic Surgery Department, Hospital de Santa Maria - Centro Hospitalar Lisboa Norte, Lisboa, Portugal.; 2 Innate Immunity and Inflammation Laboratory, Instituto Gulbenkian de Ciência, Oeiras, Portugal.; 3 Department of Biomedical Sciences and Medicine, Universidade do Algarve, Campus de Gambelas, Faro, Portugal.

**Keywords:** Bioprosthesis, Aortic Valve, Aortic Valve Stenosis, Aging, Immunology, Inflammation

## Abstract

**Introduction:**

The increase in the prevalence of aortic stenosis due to an aging population has led to an increasing number of surgical aortic valve replacements. Over the past 20 years, there has been a major shift in preference from mechanical to bioprosthetic valves. However, despite efforts, there is still no "ideal" bioprosthesis. It is crucial to understand the structure, biology, and function of native heart valves to design more intelligent, strong, durable, and physiological heart valve tissues.

**Methods:**

A comprehensive review of the literature was performed to identify articles reporting the basic mechanisms of bioprosthetic valve dysfunction and the biology of native valve cells. Searches were run in PubMed, MEDLINE® (the Medical Literature Analysis and Retrieval System Online), and Google Scholar. Terms for subject heading and keywords search included “biological heart valve dysfunction”, “bioprosthesis dysfunction”, “bioprosthesis degeneration”, and “tissue heart valves”.

**Results:**

All the relevant findings are summarized in the appropriate subsections. Structural dysfunction is a logical and expected consequence of the chemical, mechanical, and immunological processes that occur during fixation, manufacture, and implantation.

**Conclusion:**

Biological prosthesis valve dysfunction is a clinically significant process. It has become a major issue considering the growing rate of bioprosthesis implantation and improved long-term patient survival. Understanding bioprosthetic aortic valve degeneration from a basic science perspective is a key point to improve technologic advances and specifications that lead to a new generation of bioprostheses.

**Table:** Abbreviations, acronyms & symbols

AOA	= Alpha-amino oleic acid
GAG	= Glycosaminoglycans
Glut	= Glutaraldehyde
LDL	= Low-density lipoproteins
NO	= Nitric oxide
ROS	= Reactive oxygen species
SAVR	= Surgical aortic valve replacements
SVD	= Structural valve degeneration
VEC	= Valvular endothelial cells
VIC	= Valvular interstitial cells

## INTRODUCTION

Aortic stenosis is the most common primary valve disease with indication to surgery in Europe, with an increased prevalence in the last few decades due to an aging population^[1]^. In fact, more than 400,000 surgical aortic valve replacements (SAVR) are performed yearly worldwide^[2]^, contributing to a significant economic and social health issue^[3]^. It is expected that in 2050 there will be over 850,000 aortic valve replacements, worldwide^[4]^.

There are two major types of prosthetic heart valves: mechanical and biological. Randomized clinical trials^[5-7]^ comparing both types of prostheses found similar generic outcomes. However, from the published studies we can draw two important conclusions: mechanical prostheses are associated with higher rates of bleeding due to anticoagulation, while bioprostheses are associated with higher rates of reintervention due to bioprosthetic dysfunction.

Across the world, in the past decades, there has been a considerable increase in the use of bioprostheses over mechanical valves^[8-10]^, with a major shift from mechanical to bioprosthetic valves in the last 20 years. In proportion, bioprostheses increased from 40% in the 1990s to more than 80% of all implanted prosthetic heart valves nowadays^[11]^. This exponential increase in bioprosthetic valve implantation is likely related to an elderly patient population undergoing SAVR, a perceived improvement in valve durability, and a desire to avoid short and long-term anticoagulation^[8]^.

Despite the continuous efforts in the past few years, still there is no "ideal" bioprosthesis. The implementation of a prosthetic heart valve always initiates several pathophysiological processes, which can lead to structural valve degeneration (SVD) and progressive clinical deterioration. Signs and symptoms of SVD depend on the type of valve, its location, and the nature of the complication. There are several types of prosthetic dysfunction, ranging from structural/non-structural deterioration of the valve, thrombosis, and endocarditis^[12]^.

For the past 50 years, glutaraldehyde (Glut) has been the most used chemical product and is currently widely used to preserve and stabilize biological prosthetic tissues. Glut is responsible for chemical cross-linking, improving the material’s stability and reducing antigenicity. Bioprosthetic heart valves show several histological differences from native heart valves, being unable to remodel and repair. The manufacture process of prostheses is also crucial, especially regarding fixed configuration of the pericardial valves and the pressure used in tissue fixation.

Recently, research has brought new ideas about the inflammatory and immunological roles in bioprosthetic dysfunction, describing the immunological rejection and the inflammatory state also as causes of failure of bioprostheses.

In terms of durability, new-generation bioprostheses may have promising results^[11]^. The reported durability is excellent, with rates of reintervention due to failure of the bioprosthesis of 2% to 10% in 10 years, 10% to 20% in 15 years, and 40% in 20 years^[13,14]^. However, these findings do not show the true rates of deterioration of bioprostheses. Some studies have identified higher rates of structural deterioration, including hemodynamic changes, in up to 10% and 30% of patients five and 10 years after surgery, respectively^[11]^.

The significant increase in the use of aortic bioprosthesis will inevitably lead to a proportionally rising number of patients diagnosed with prosthetic dysfunction in the next decade. This should stimulate cardiac surgery centers and medical prosthesis manufacturers to understand all underlying mechanisms. This article aims to review the most debated topics on the pathophysiology of aortic bioprosthetic dysfunction, exploring the biological grounds on the chemical, mechanical, and inflammatory contribution to better understand the most recent innovations in this field.

## METHODS

A comprehensive review of the literature was performed to identify articles reporting the basic mechanisms of bioprosthetic valve dysfunction and the biology of native valve cells. Searches were run in March 2020 in the following databases: PubMed, MEDLINE® (the Medical Literature Analysis and Retrieval System Online), and Google Scholar. Terms for subject heading and keywords search included “biological heart valve dysfunction”, “bioprosthesis dysfunction”, “bioprosthesis degeneration”, and “tissue heart valves”. There was no patient involvement in this study, so ethic board’s approval was not required.

## RESULTS AND DISCUSSION

### Heart Valves

Natural heart valves are unique structures, adapted to allow unidirectional and nonobstructive blood flow. They are biologically dynamic structures, naturally designed to avoid regurgitation, thromboembolism, trauma to their molecular and cellular structures, or disruptive stress. To understand the pathophysiology of biological prosthetic valve dysfunction it is crucial the understanding of the structure, biology, and function of native heart valves.

In the embryonic developing heart valve, epithelial-to-mesenchymal transition occurs when cells from the endocardium differentiate into mesenchymal cells and migrate into the cardiac jelly that forms the pre-valve cardiac cushions^[15]^. These cushions are rich in glycosaminoglycans (GAG), such as hyaluronic acid, and signaling molecules responsible for further development of heart valves^[1,2]^. The cardiac cushions are responsible for the complex regulation of extracellular matrix proteins, producing an intricate and functional structure^[16]^. Although the immature heart valve produces its own extracellular matrix *in utero*, their development is only completed in the postnatal life^[1,3]^. Characterizing the embryonic progenitors of heart valve cells and development processes is important to understand basic pathogenesis in valvular disease. Exploring cellular and molecular pathways in valvular disease will eventually allow designing more intelligent and physiological heart valve tissues.

Development of heart valves leads to a layered, structured, and highly specialized complex structure of adapted cells and extracellular matrix^[17,18]^. Their configuration will allow heart valves to assure its highly specified function and maintain their strength and durability despite the regular and repetitive stress and strain. Heart valves also need to have elements that assure permanent repair and remodeling. Although there are four different heart valves with different configurations and functions, all of them have a similar layered patter of cells.

**Fig. 1:**
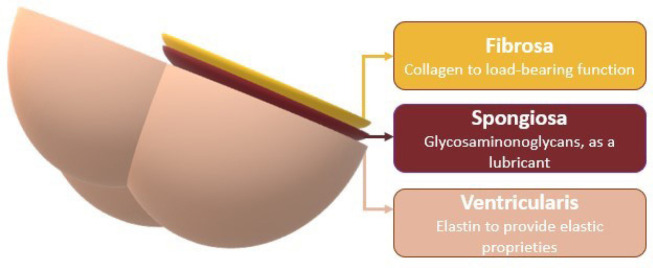
Schematic image of the three cardiac valve leaflets.

Valve leaflets are mainly constituted by collagen type I and III, proteoglycans, GAG, and elastin^[19]^. The leaflet has three layers, each one with an important microstructure (Figure 1): 1) *fibrosa*: the outflow surface, with circumferentially and densely aligned packed collagen fibers, to enable a load-bearing function during diastole^[20]^; 2) *spongiosa*: the middle layer, which is rich in GAG and acts as a lubricant between the two other layers^[20-22]^; and 3) *ventricularis*: the inflow surface, predominantly with elastin to provide elastic proprieties^[20,21]^. The arrangement and configuration of the extracellular matrix is responsible for the changes in shape and dimensions throughout the cardiac cycle.

Two major types of cells are present in the leaflets: the valvular endothelial cells (VEC) and the valvular interstitial cells (VIC). Both groups of cells synergize to maintain the normal function of the valves.

### Valvular interstitial cells

VIC represent a crucial and heterogeneous cell population through the leaflet, distributed in all layers^[23]^. They are the most abundant cell type in the heart valves and resemble, among others, fibroblasts and smooth muscle cells. VIC represent a dynamic population responsible for the synthesis of extracellular matrix and matrix-degrading enzymes. Thus, they regulate and remodel collagen and other essential components to assure the continuous valve repair^[23]^. The activation of VIC (including the production and secretion of matrix) is regulated by mechanical stretch, local cellular signaling (*e.g*., interaction with other types of cells from heart valves such as VEC), microstructural factors, and hemodynamic environments^[24]^. It has also been theorized that VIC contraction, in response to environment stimuli, may facilitate cell-to-cell communication and act as a role in maintaining leaflet homeostasis^[25]^. The ability to answer to the surrounding environment makes VIC highly plastic, with at least five distinct phenotypes described, from a quiescent to an activated form (the five distinct VIC phenotypes include embryonic progenitor endothelial/mesenchymal cells, quiescent VIC, activated VIC, post developmental/adult progenitor VIC, and osteoblastic VIC)^[23]^.

Although it may be interesting to explore all phenotypes, the quiescent and the activated forms are the most relevant in this context. VIC in adult valves are quiescent, without activity, with fibroblasts characteristics^[26,27]^. The plasticity of VIC is important for development but plays also an important role in pathologic processes^[19]^. VIC plasticity is regulated by multiple factors, such as environmental factors and host factors, as age.

In the disease states, VIC progress from the quiescent fibroblast-like phenotype to a contractile form, with enhanced production and secretion of extracellular matrix, cytokines, proteases, and growth factors^[9,10]^. VIC progression has direct consequences on the heart valve function, as it has been shown that VIC contraction have measurable effects on leaflet stiffness^[28]^. When the activation persists and is prolonged, VIC can differentiate into osteoblast-like cells (the osteoblastic VIC phenotype), leading to calcified nodule formation and valve calcifications^[29]^. However, activation of VIC is not a definitive process, as it can be reversibly modulated^[29]^.

### Valvular endothelial cells

VEC represent the cell layer that covers the leaflets, in line with the endothelium from the entire cardiovascular system. However, VEC are phenotypically different from other cardiovascular endothelial cells, probably due to the fact that they interact with VIC to maintain the integrity of valve tissues and, potentially, also to mediate disease^[26]^. They are highly specialized endothelial cells: usually endothelial cells in vascular tissues are typically elongated in the direction of blood flow, but on the leaflets they have a circumferential arrangement to support leaflet stresses and mechanical forces^[30]^. As all endothelial cells, VEC are crucial to function as a barrier, regulating interactions between blood flow and VIC, including metabolic and inflammatory processes^[31]^.

VEC may also exhibit different phenotypes depending on their location on the valve leaflets^[32]^. Endothelial cell production of nitric oxide (NO) is an important crosstalk to maintain VIC quiescent, and endothelial injury has been proposed to be an initiating factor for calcified aortic valve disease^[33]^. VEC are also responsible for the non-thrombogenic proprieties of the leaflets, playing an important immune and inflammatory role^[34]^. VEC are also capable of responding to mechanical stimuli, with a flow-mediating mechanotransduction process being responsible for the activation of protective or pathological pathways^[35]^.

### Heart Valves as a Functional System

Understanding the molecular and cellular components of the heart valves will allow us to understand the biomechanical features of the valves.

The aortic valve is the most frequently diseased and studied valve and it is the perfect paradigm to represent the complex and highly specialized extracellular matrix configuration of heart valves. During diastole, the aortic valve leaflets stretch to avoid blood backflow. The change in its configuration is predominantly dependent on collagen, with directional realignment and crimping of the fibers. The collagen orientation determines tissue compliance to tensile stress. This complex fiber architecture is very sensitive to pressure, and very low pressures at the beginning of the cardiac cycle are capable of inducing collagen fibers arrangements, with loss of the *fibrosa* corrugations and collagen crimps^[15-18]^. Mechanical functions of collagen also include limitation of cuspal stretching to avoid prolapse (achieved mainly by the strained and aligned collagen in the *fibrosa*)^[23]^.

During systolic valve opening, the tissue becomes relaxed, with the elastin from the *ventricularis* layer recoiling to make the cusp retracted again^[20,26]^. In this phase of the cardiac cycle, fibers have a random directional distribution and crimps of collagen fibrils are restored^[23]^.

All these continuous and coordinated alterations in valve configuration depend on the quality and quantity of its components, such as collagen, elastin, and GAG. Indeed, they are the major determinants of the functional mechanisms and long-term durability of the valve. It has already been showed that the cell source used in bioprosthesis is important for long-term durability since the lack of cells in bioprosthesis has been shown to be the main source of failure for implants^[26]^. That is the reason why all the previously described components are essential to maintain valve homeostasis. Thus, cell source and function are important components of heart valve tissues, as they depend on the viability and function of active valve cells^[23]^.

### Bioprosthetic Dysfunction

Biological heart valves consist of fixed or decellularized human or animal (usually porcine or bovine) tissue, attached or not to a stent (without a stent in the stentless prosthesis) and a sewing ring (without sewing ring in the sutureless prosthesis). Structural dysfunction is a logical and expected consequence of the chemical, mechanical, and immunological processes that occur during fixation, fabrication, and implantation.

Beside all the technological development in the past decades, bioprostheses only replicate native heart valve structure. In bioprostheses, 1) the cusps are constituted by subendothelial connective tissue; 2) the cusps are locked in a static geometry; and 3) they have nonviable cells due to the fixation and cross-linking processes^[21,22]^. They lack the functional capacities of heart valves and the preservation techniques severely decrease functional activity of the natural matrix^[17]^. Thus, they are not able to maintain the normal remodeling processes.

Glut, a currently widely used chemical to preserve and stabilize biological prosthetic tissues, is an aldehyde with fixative and preservative functions^[35]^, which was firstly introduced in 1963 by Sabatini et al.^[22]^ as a fixative for electronic microscopy. Glut is responsible for the chemical cross-linking (creation of covalent chemical bonds to stabilize tissues and terminate any ongoing biochemical reactions), enhancing material stability, and reducing antigenicity^[29,30]^. However, Glut is also partly responsible for prosthetic dysfunction, directly because of its toxicity and indirectly through the processes described ahead.

**Fig. 2:**
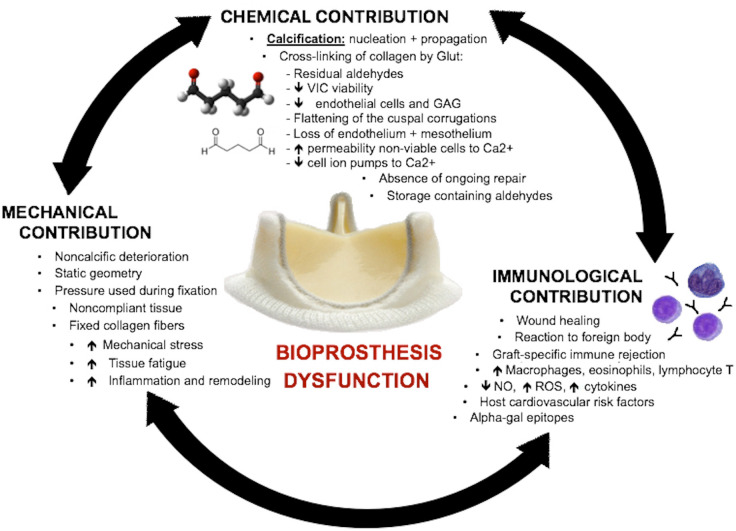
Representation of the chemical, mechanical, and immunological factors that contribute to prosthetic valve dysfunction. GAG=glycosaminoglycans; Glut=glutaraldehyde; NO=nitric oxide; ROS=reactive oxygen species; VIC=valvular interstitial cells

Valve degeneration is a multifactorial process, including chemical, mechanical, and immunological factors (Figure 2). The contribution of each mechanism remains poorly understood but represents an active and attractive field of investigation. The common endpoint of all these processes is calcification and degradation, culminating in the failure of the structure with bioprosthetic dysfunction.

### Chemical Contribution

Glut cross-linking make tissues biocompatible and nonthrombogenic, while maintaining anatomic integrity, leaflet strength, and flexibility^[36]^. Its preference among aldehydes is related to its availability, low price, quick action, and capacity to react with a large number of amino acids^[36]^.

Glut is responsible for the effective cross-linking of collagen, the most common structural component of the valves. It forms covalent bonds by the formation of Schiff bases (reaction of an aldehyde group with an amino group of lysine or hydroxylysine) and/or by aldol condensation between two adjacent aldehydes^[37]^. Cross-linking increases tissues durability, reducing resistance to proteolysis of the cross-linked proteins. However, after Glut fixation, residual aldehydes remain expressed on the tissue surface and may act as calcification locations.

Although its action is essential for valve preservation and to eliminate cellular components to reduce tissue immunogenicity, the induced chemical reactions are probably the most important part of bioprosthetic valve degeneration. After Glut fixation, VIC lose their viability^[38]^. However, some studies have showed that Glut retains many of the viscoelastic proprieties of the collagen^[36]^, with hemodynamic proprieties of the prostheses similar to those of living tissues^[36]^.

Additionally, as part of the fixation and fabrication process, the cellular content of the tissues is modified, with loss of endothelial cells, loss of interstitial cell viability, and interstitial cell degeneration^[39]^. Indeed, bioprosthetic heart valves show several histological differences from native heart valves, with flattening of the cuspal corrugations, loss of the endothelium or mesothelium surfaces, disruption of interstitial cells, and loss of GAG^[38]^. With the loss of interstitial cells viability, the mechanical proprieties and durability of the valve depend primarily on the quality of the collagen and the remaining viscoelastic proprieties are not enough to avoid valve tissue degeneration.

Schoen et al.^[39]^ described the calcification process as having two phases: nucleation (or initiation) and propagation. One important change to initiate calcification is the abnormal extrusion of calcium ions from the nonviable cells. Cross-linking to proteins of the cellular membrane alters their proprieties, resulting in a different permeability in the non-viable cells. Additionally, there is a reduction of the functional transmembrane ion pumps and an increased permeability to calcium ions that contribute to the onset of calcifications^[40]^. Usually, calcium concentration is 1,000 to 10,000 times lower in the cytoplasm due to the healthy ion pumps that carry calcium out of the cells. With deregulated calcium levels inside the cells, cellular membranes and other intracellular structures bind calcium and serve as nucleators for calcification. Indeed, calcification seems to start predominantly at the cell membranes and other intracellular structures rich in phospholipids, while the loss of proteoglycans may enhance this phenomenon by removing calcification inhibitors^[41]^. Glut also reacts directly with intracellular structures, predisposing to calcification in the presence of high intracellular calcium levels^[42]^. The debris of interstitial cells that remain in valve tissue also serve as initiation sites for calcification.

Collagen and elastic fibers can also serve as nucleation sites, independent of cellular components. One important difference is that calcification of collagen requires cross-linking alterations, while calcification of elastin occur independently of cross-linking^[43]^.

After the nucleation, calcification is influenced by all the metabolic and pathologic changes in calcium and phosphorus metabolism, with calcium-enriched crystals growing to eventually culminate in prosthetic malfunction (propagation phase).

Another important characteristic of native heart valves is their remodeling and reparation capacity. In biological prosthesis, the fixed and nonviable tissue is incapable to maintain the ongoing repair, and every damage do the extracellular matrix is cumulative. Moreover, endothelial cells are denuded or absent and adjacent smooth muscle cells might proliferate and migrate freely to the non-endothelialized valve surface^[44]^, also contributing to valve dysfunction.

The dynamic role of native valve cells and their importance for the durability of bioprosthesis is now recognized, and new strategies of repopulation and regeneration have been proposed to minimize the problem. Repopulation defines the process of using a clean connective tissue matrix valve, repopulated with the recipient cells, before or after prosthesis implantation^[45]^. A completely “self-populated” prosthesis would maintain tissues invisible, avoiding host reaction to the graft. Although this technology reached clinical practice, the results were not as good as expected. Regeneration involves the implantation of a remodeling matrix with the proteins and cells of the recipient that can resemble the dynamic changes of native heart valves^[45-49]^, but it remains in the pre-clinical development.

Storage is another important issue regarding bioprosthetic valve dysfunction. Several bioprostheses are stored in liquids containing aldehydes, which are toxic and a source for calcification, as abovementioned. Regardless fixation and production procedures with a reduced aldehyde content, tissues are exposed once again to deleterious free aldehydes when they are stored in aldehyde enriched solutions. Even pre-implant rinsing does not guarantee complete removal of toxic aldehydes with such storage solutions, and new technologies regarding storage are also an active field of research.

### Mechanical factors

In the past, heart valve dysfunction was assumed as a degenerative and passive deposition of calcium crystals. However, it has been established that biological prosthetic valve dysfunction is a very dynamic and progressive process, including noncalcified deterioration with important mechanical and environmental contributions.

As part of the fabrication model of bioprosthesis, the structure is fixed in a static geometry. The collagenous network is locked into a single configuration of one phase of the cardiac cycle, inhibiting the normal extracellular matrix readjustments of the valve during the cardiac cycle^[39,40]^. The collagen crimps and corrugations are preloaded in a defined configuration similar to that of a closed valve^[48]^. Thereby, functional and normal cardiac cycle stresses have to be absorbed by the noncompliant and fixed collagen fibers^[48]^. Having a fixed configuration will produce damage in pericardial valves during closure (in porcine valves, mostly during opening but also during closing), inducing repetitive accumulation of mechanical stress with increased tissue fatigue^[39,40]^. Leaflets should display anisotropic proprieties, especially regarding strain in the circumferential and radial directions. It has been established that non-physiological strain leads to pathologic processes by deregulation of inflammation and remodeling, leading to calcification^[18]^.

The pressure used during fixation is also important for the mechanical changes induced in the tissues, as it determines the stress-strain relation of the tissue strips^[50]^. Low pressures have better mechanical proprieties and most of the bioprostheses used nowadays, such as Edwards Lifesciences® Magna Perimount and LivaNova® Perceval Plus, are manufactured with low pressure fixation^[51]^. However, studies have shown that as low as 2-4 mmHg are sufficient to induce significant changes in collagen compliance^[48]^, so zero-pressure methods have been studied. Although in theory zero pressure would offer a new approach to reduce biological prosthetic dysfunction, the technique has not achieved clinically significant results^[52]^.

Although Glut-related dysfunction is more associated with calcification, it also induces mechanical alterations to the valve tissue. It has been shown that Glut alters the stress-strain curves of strips of bovine and porcine valve tissues^[50]^. During the fixation process there is also a considerable loss and incomplete stabilization of the GAG^[53]^, which are responsible for the viscoelasticity and accommodation of the cuspal layers. GAG have an essential role in absorbing compressive loads, modulating shear stress, and avoiding tissue buckling, and they may be important in mechanical abnormalities that lead to valve dysfunction.

The chemical and mechanical changes are possible synergetic, since changes in tissue configuration can induce stress and fatigue (especially in flexion lines of the cusps) that can expose and disrupt collagen, initiating the calcification process. On the other hand, calcifications can also induce structural changes and stiffness that will eventually lead to more mechanical changes and damage.

### Immunological factors

Recently, research has brought new insights on the inflammatory and immunological roles in bioprosthetic dysfunction, describing immune rejection also as a cause for bioprosthetic failure^[40,51]^.

In all biological processes, immune responses have several grades, from a physiological to pathologic state. Even in the setting of a bioprosthesis, inflammation can be divided into several types: 1) the postsurgical normal wound healing; 2) nonspecific innate inflammatory reaction to a new foreign body; and 3) immune-mediated rejection and/or inflammation^[54-56]^. We will focus on points 2 and 3, since the normal postsurgical wound healing is not in the scope of this review.

Despite all the fixation and processing procedures, Glut decreases but not entirely eliminates the antigenicity of tissue valves^[57]^. Collagen matrix (with cell debris and necrotic products) elicits a strong nonspecific inflammatory response, including infiltration by macrophages and eosinophils, followed by a lymphocyte T response^[58]^. Persistent antigenicity of bioprosthesis has been shown to continually stimulate graft-specific adaptive immune reactions with important biomaterial dysfunction^[58]^. The dysfunctional endothelial layer may also contribute to maintain the inflammatory status, probably through the reduced NO production and increased generation of reactive oxygen species and inflammatory cytokines^[59]^.

Glut itself also causes some degree of inflammation^[60]^. Additionally, inflammation and calcification are also linked. It has been shown that calcification is associated with the amount of inflammation, as lymphocytes and macrophages produce osteopontin (an important cytokine in the calcification process)^[60]^. Inflammatory and fibroblast signaling contribute to a pro-osteogenic environment (with the activation of quiescent VIC to the osteogenic VIC phenotype) and remodeling process, predisposing to dystrophic calcification^[61]^.

Host cardiovascular risk factors may also contribute to the inflammatory environment. Studies have demonstrated that some risk factors such as dyslipidemia, diabetes, or metabolic syndrome may modulate bioprosthetic degeneration through inflammation^[46,47]^. Analyses from the explanted prosthesis have also revealed that explants are usually infiltrated by oxidized low-density lipoproteins (LDL), beside inflammatory cells^[62]^. Indeed, patients with SVD have a tendency of higher triglyceride levels and high levels of small, dense LDL, which are associated with prosthetic dysfunction^[62]^. Moreover, Mahmut et al.^[63]^ have shown that lipoprotein-associated phospholipase A2 (or Lp-PLA2), an enzyme that produces pro-inflammatory substances from LDL, is an independent predictor of SVD.

Another important factor that has recently been associated with bioprosthetic valve dysfunction is alpha-gal. Alpha-gal (or galactose-alpha-1,3-galactose) is a carbohydrate found in most mammalian membranes, but not in humans. Humans normally display anti-gal antibodies due to antigenic stimulation, representing an important barrier to xenotransplantation^[64]^. Yet, alpha-gal epitopes are present in bioprostheses, even in decellularized tissues. It has been shown that the implantation of bioprostheses induce a specific immune reaction to the alpha-gal antigen, with the production of anti-alpha-gal antibodies^[64]^. The interaction between the circulating anti-alpha-gal antibodies and calcification of bioprosthesis has been established^[50,51]^, but the contribution to long-term dysfunction is not yet completely understood. However, basic research continues and recent studies have shown that engineered pericardial tissue from alpha-gal-deficient pigs calcifies less in animal models^[65]^. Tissue valve investigation continues in order to design new tissues with less alpha-gal epitopes, and a genetically modified pig with no expression of alpha-gal has already been generated, but more studies are needed^[66]^.

### New Generation of Aortic Bioprosthesis: Technology Specifications

One important point in tissue valve engineering is the ability to develop new biological prosthesis with less static configurations and more biologically active tissues. So far, recent technologies have achieved better fixation and storage procedures (Table 1), but all of them fail to mimic biological activity of heart valve cells. Recently, two new bioprostheses (Inspiris® from Edwards Lifesciences® and Perceval Plus® from Livanova®) with two novel tissue treatments (Resilia® and FREE®, respectively) have been launched expecting less calcification and improved tissue durability.

**Table 1:** Comparison of chemical, mechanical, and immunological advances between bioprosthetic valves.

		Valve	Manufacturer	Anticalcification treatment	Chemical	Mechanical	Immunological	References
Non-stented	Porcine	Toronto SPV®	Abbott®	Linx AC® (ethanol treatment)	- Ethanol breaks down cellular membranes, inhibiting calcium nucleation - Increased lipids’ extraction - Reduced free aldehydes - Minimizes cholesterol uptake	- Stabilizes leaflet collagen - Enhances cuspal resistance to collagenase	---	-82,83
		Freestyle®	Medtronic®	Alpha-amino oleic acid (AOA)	- Mitigates calcification - Diminishes Ca^2+^ diffusion	- Physiologic fixation process: zero pressure with preservation of structure and leaflet function - Soft and flexible leaflets protecting from cycle fatigue - Stentless configuration: larger postoperative effective orifice area	---	-84
	Bovine	Solo Smart®	LivaNova®	Homocysteic acid	- Detoxification process with homocysteic acid (neutralizes residues of unbound glutaraldehyde) - Improved endothelial cell proliferation - Aldehyde-free storage	- No fabric reinforcement - No stent, no synthetic material - One single suture for all the leaflets	---	-85,86
Stented	Conventional - Porcine	Hancock II®	Medtronic®	T6 (sodium dodecyl sulfate)	- Inhibits calcification of collagen - Higher removal of phospholipids by T6	- Acetal homopolymer stent with flexible and lower profile leads to higher stress absorption - Low pressure fixation process minimizes septal muscle shelf	---	(87-89)
		Mosaic®	Medtronic®	AOA	- Surfactant washes and removes phospholipids - Mitigates calcification - Diminishes Ca^2+^ diffusion - AOA forms covalent bonds with free aldehydes	- Next generation flexible polymer stent allows higher absorption of stress - Physiologic fixation process minimizes the stress (zero-pressure fixation) - Soft and flexible leaflets protecting from cyclic fatigue)	---	-47,9
		Epic®	Abbott®	Linx® anticalcification	- Inhibition of Ca^2+^ nucleation - Higher lipids’ extraction - Reduced free aldehydes - Minimizes cholesterol uptake - Lower cusps water content	- Flexible polymer stent - Stabilizing leaflet collagen - Enhanced material stability (in vitro increased resistance to collagenase digestion)	---	-82,83
	Conventional - Bovine	Perimount®	Edwards Lifesciences®	XenoLogiX® treatment	- Alcohol and surfactant extract phospholipids from pericardial tissue after glutaraldehyde fixation (Bio-Burden reduction) - 8-10 log reduction in microbial levels after sterilization	- Flexible cobalt-chromium alloy stent reduces leaflet stress - Leaflets matched for thickness and elasticity to reduce stress distribution	---	-91,92
		Magna Ease®	Edwards Lifesciences®	ThermaFix®	- Phospholipids extraction (as XenoLogiX® treatment) - Mild heat treatment to remove unstable glutaraldehyde moieties - Reduced tissue acid levels, with less potential binding sites for calcium - Maintenance of collagen and elastin structures	- Flexible cobalt-chromium alloy stent reduces leaflet stress - Three independent pericardial leaflets	---	-92
Stented	Conventional - Bovine	Inspiris Resilia®	Edwards Lifesciences®	RESILIA®	- Functional group (aldehydes) stable capping, glycerolisation, and ethylene oxide sterilization - Free aldehyde groups blocking in the tissue - Dry glutaraldehyde-free storage (glycerol)	- Valve structure is built over 3 semi-rings instead of 1 allowing future valve-in-valve	---	-93,94
		Trifecta GT®	Abbott®	Linx® anticalcification Glide Technology	- Inhibition of Ca^2+^ nucleation - Higher lipids’ extraction - Reduced free aldehydes - Minimizes cholesterol uptake - Stabilizes leaflet collagen	- Soft compliant sewing cuff - High-strength titanium stent to reduce stress on leaflets and allowing a cylindrical opening during systole - Pericardial-covered stent to reduce risk abrasion - Computer controlled fiber orientation	---	(82,95,96)
		Avalus®	Medtronic®	AOA	- Surfactant washes and removes phospholipids - AOA forms covalent bonds with free aldehydes - Slows movement of Ca^2+^ on tissue - Stored in glutaraldehyde solutions to induce covalent bonds on remaining free aldehydes	- Laser cut leaflets matched for thickness and deflection for a consistent performance - Two-part polymer frame minimizes stress - Uniaxial fixation	---	-97
	Rapid deployment - Bovine	Intuity Elite®	Edwards Lifesciences®	ThermaFix®	- Phospholipids' extraction (as XenoLogiX treatment) - Mild heat treatment to remove unstable glutaraldehyde moieties - Reduced tissue acid levels, with less potential binding sites for calcium - Maintenance of collagen and elastin structures	- Three independent leaflets matched for thickness and elasticity - Flexible alloy wireform reduces shock during cardiac cycle - Stainless steel frame	---	-92
		Perceval Plus®	LivaNova®	FREE®	- Reduces phospholipids content in the tissues (alcohol mixture)	- Elastic structure allows stress absorption at the commissures (double sheet design)	- Coated by a thin layer of turbostratic carbon (CarboFilm®), increasing biocompatibility and encouraging endotheliazation	(98)
					- Highly effective neutralization of aldehydes (post-sterilization amino acid treatment), with a very low level of aldehydes	- Stent adapts to the movements of the aorta during cardiac cycle		
					- Completely aldehyde-free storage	- Inflow ring expands to accommodate valve-in-valve		
					- Removal and neutralization of unbound glutaraldehyde	- Physiologic fixation to collapse without crimping collagen fibers		

Perceval Plus® is a bovine pericardial heart valve with a novel tissue treatment to reduce calcification: the FREE® treatment. FREE® treatment uses an alcohol mixture for phospholipids removal, combined with a post-sterilization amino acid treatment for the neutralization of unbound aldehydes, and final storage with an aldehyde-free solution^[67]^. FREE®-treated tissues have a reduced content of phospholipids up to 96%, comparing to Glut-treated tissues, combined with a significant improvement in the removal and neutralization of unbound Glut^[67]^. Tissues have less propensity to mineralization while maintaining the same mechanical and biochemical performance and stability of the conventional Glut-treated prosthesis^[67]^. In theory, FREE® treatment is an effective strategy to reduce bioprosthetic dysfunction. However, long-term outcomes in humans are still unknown.

Resilia® is also a new tissue preservation technology, that uses stable functional group capping, ethylene oxide sterilization, and preservation by glycerolization^[68]^. This innovative technology does not avoid Glut use but reduces phospholipids content and residual chemicals from the valve tissues. There are also differences considering the storage of the tissues, using dry storage, which reduces the contact of the prosthesis with aldehyde-enriched solutions.

Despite all these new and exciting tissue treatments to prevent calcification, we should keep in mind that it will take a long time to have the 15-20-year outcomes we now have for conventional Glut-treated tissues. Moreover, all these strategies are based on the reduction of free aldehyde groups and phospholipids content, reducing the chemical effects of Glut. As previously discussed here, chemical changes are just part of the complex process of biological prosthetic valves dysfunction, and new strategies to address mechanical and immunological changes in valve tissues must be addressed.

## CONCLUSION

Biological prosthesis valve dysfunction is a clinically significant process. It has become a major issue and a hot topic considering the growing rate of bioprosthesis implantation and improved long-term patient survival.

Although the biological prosthesis has important advantages over mechanical ones, durability is the major limitation of their implantation. The design and development of new biological bioprostheses must reproduce the structure, and, more important, the biology of native heart valves. Understanding the complex biologically functional and dynamic system of the heart valves will elucidate how bioprostheses can match their natural behavior. Every surgeon should be aware of their biological complexity to understand and discuss new tissue valve technologies to provide patients heart valves with improved durability and better performance.

Biological heart valves have been used for more than 50 years, but bioprosthetic dysfunction remains a challenging and intriguing field, with an enormous quantity of work and research ahead in each of its subjects. SVD remains an interesting field because the pathophysiology of SVD is not yet completely known. Only in the past few years, studies have unveiled new biological pathways. And that is the reason why basic research on the pathophysiology of SVD remains crucial. We will only achieve new and efficient tissue development if we deepen our knowledge of the biological processes of native heart valves. Fortunately, research on tissue heart valves is now wide and covers several different fields, such as 1) the effects of Glut fixation, 2) non-Glut fixation, 3) mechanisms of calcification and non-calcification dysfunction, 4) anticalcification approaches, 5) biology of the valvular cells, and 6) tissue-engineered valves^[39]^.

Concluding, biological heart prosthetic dysfunction is a complex and multifactorial process, with biological, chemical, mechanical, and immunological factors. Chemical changes caused by Glut and the consequent bioprosthetic calcification have been the main target of tissue valve development. However, as pathophysiology has been explored, with more detailed knowledge on cell structure and function and on the inflammation associated with bioprosthetic valve implantation, new questions arise. SVD remains a challenging field of research and novel interventions and developments need to focus on strategies that target the cell and immune events responsible for degeneration and rejection of the tissues. Moreover, new tissues must keep some of the biological proprieties of native heart valves, since valve changes in conformation with the cardiac cycle and regeneration processes are essential, but many times forgotten aspects of SVD.

**Table:** Authors' roles & responsibilities

TRV	Substantial contributions to the conception or design of the work; interpretation of data for the work; final approval of the version to be published
RMP	Substantial contributions to the conception or design of the work; interpretation of data for the work; final approval of the version to be published
FF	Substantial contributions to the conception or design of the work; interpretation of data for the work; final approval of the version to be published
NCG	Revising the work; final approval of the version to be published
RF	Revising the work; final approval of the version to be published
AN	Revising the work; final approval of the version to be published
